# Modulation of IRAK4 as a therapeutic strategy against monosodium urate- and xanthine-induced inflammation in macrophages and HepG2 cells

**DOI:** 10.3389/fimmu.2025.1744393

**Published:** 2026-01-08

**Authors:** Sadiq Umar, Huan T. Chang, Mark Maienschein-Cline, Sriram Ravindran

**Affiliations:** 1 Department of Oral Biology, University of Illinois Chicago, Chicago, IL, United States; 2 Division of Rheumatology, University of Illinois Chicago, Chicago, IL, United States; 3 Jesse Brown VA Medical Center, Chicago, IL, United States; 4 Research Informatics Core, Research Resources Center, University of Illinois, Chicago, IL, United States

**Keywords:** HepG2, inflammation, IRAK4 inhibitor, macrophages, NF-κB signaling, monosodium urate crystal, xanthine

## Abstract

**Background:**

Interleukin-1 receptor-associated kinase 4 (IRAK4) is a pivotal mediator of Toll-like receptor (TLR) and interleukin-1 receptor (IL-1R) signaling, critically involved in innate immune activation and pro-inflammatory cytokine production. Dysregulated IRAK4 activity contributes to chronic inflammation in both immune and non-immune cells. In this study, we evaluated the immunomodulatory potential of a selective IRAK4 inhibitor on monosodium urate (MSU) crystal-stimulated macrophages and xanthine-challenged HepG2 cells to assess its therapeutic potential.

**Methods:**

Human peripheral blood mononuclear cells (PBMCs) were pretreated with 1 µM IRAK4 inhibitor (IRAK4i) overnight, followed by stimulation with 100 µg/mL of MSU for either 30 min or 24 h. Conditioned medium was collected for ELISA and RNA for qPCR to quantify pro- and anti-inflammatory factors. Cell lysates were prepared to analyze various TLR/IL-1β signaling proteins, including phosphorylated IRAK4, P38, ERK, and JNK. Phagocytosis was assessed using a Vybrant™ phagocytosis assay kit in PBMCs. HepG2 cells were also utilized and pretreated with 1 µM of IRAK4i overnight, followed by stimulation with 2.5 mM of xanthine for 24 h to assess the expression of pro-inflammatory cytokines and xanthine oxidoreductase.

**Results:**

Primary macrophages and HepG2 cells were treated with a potent IRAK4 inhibitor in the presence and absence of MSU or xanthine. In macrophages, IRAK4 inhibition significantly reduced the expression of TNF-α, IL-6, and IL-1β at both mRNA and protein levels while promoting polarization toward an anti-inflammatory (M2-like) phenotype alongside reduced activation of the nuclear factor-κB (NF-κB) and MAPK pathways. In HepG2 cells, IRAK4 blockade attenuated xanthine-induced expression of xanthine dehydrogenase and pro-inflammatory cytokines.

**Conclusion:**

These findings demonstrate the dual anti-inflammatory effect of IRAK4 inhibition in both immune and hepatic cells and suggest a promising strategy to mitigate inflammation in gout.

## Introduction

Gout is presently recognized as the common root of inflammatory arthritis. Its incidence and prevalence are on the rise in both developed and developing nations (1–4). The inflammatory response in gout is triggered by the deposition of uric acid crystals in the articular joints of individuals with hyperuricemia, leading to severe inflammation and excruciating pain. In the body, purine metabolism produces uric acid as a byproduct, and its imbalance between production and excretion or reabsorption results in elevated levels of uric acid in bodily fluids (5–7). The incidence of gout is further increased in individuals who consume red meat and alcohol (8). Uric acid deposition can also lead to complications such as renal dysfunction, cardiovascular diseases, and diabetes (9–12).

Research has shown that the initial inflammatory response in gout involves articular resident macrophages within the joint space phagocytosing monosodium urate (MSU) crystals. These engulfed MSU crystals interact with Toll-like receptors (TLRs), especially TLR2/4 (13–16). These TLRs’ recognition of MSU leads to the activation of nuclear factor-κB (NF-κB) and the NLRP3 inflammasome, resulting in the activation of caspase-1 and the processing and secretion of interleukin-1β (IL-1β). IL-1β, along with other pro-inflammatory cytokines such as IL-6, TNF-α, and IL-8, promotes neutrophil invasion (17–22). Infiltration of these neutrophils contributes to joint damage through the release of various mediators, including reactive oxygen species, cytokines, chemokines, proteolytic enzymes, and prostaglandin E2 (PGE2), which leads to the degradation of cartilage (23–25).

Interleukin-1 receptor-associated kinase 4 (IRAK4) is a serine–threonine kinase in the TLR/interleukin-1 receptor (IL-1R) signaling cascade (26). Upon stimulation, the cytoplasmic receptor domain binds to the intracellular adaptor protein MyD88, resulting in the assembly of MyD88 and IRAK family (IRAKs 1–4) into a complex known as the Myddosome. IRAK4 initiates the production of pro-inflammatory cytokines via the activation of transcription factors (NF-κB and AP-1) (5, 13, 24, 27–29). Our studies have shown that the onset of rheumatoid arthritis (RA) is abrogated by the inhibition of IRAK4 in TLR7-induced inflammation in macrophage and fibroblast cell-based and *in vivo* models of joint inflammation (30–32).

Previous studies have shown that TLR2/4 activation amplifies inflammation in myeloid cells through NF-κB signaling, and the expression of these receptors correlates with disease activity in gout (33). Notably, TLR2/4 levels decrease in patients during remission compared to active flare (34). Given this pattern, we explored whether targeting IRAK4—a common downstream mediator of TLR signaling—could attenuate the inflammatory response associated with gout. Our findings support this approach, showing that IRAK4 blockade dampens key inflammatory pathways activated by both MSU and xanthine.

## Methods

### Gene expression analysis from gout patients

The single-cell RNA sequencing dataset GSE211783 submitted by Hanjie Yu et al. (34) for gout flare and gout remission was accessed via the web interface (https://www.ncbi.nlm.nih.gov/geo/query/acc.cgi?acc=GSE211783) to evaluate the expression of TLRs and cytokines. This cohort consisted of gout patients who were older than 18 years and met the 2015 American College of Rheumatology (ACR) and European League Against Rheumatism (EULAR) classification criteria.

### Cell culture

#### Human myeloid cells

The study was approved (approval number: 2021-1435) by the Institutional Ethics Review Board, University of Illinois at Chicago (UIC). Healthy samples were purchased from Oklahoma Blood Institute (Oklahoma City, OK). Peripheral blood mononuclear cells (PBMCs) were isolated from healthy donors using Ficoll-Paque-based density centrifugation, as described earlier (30). Human monocytes from healthy donors were differentiated into macrophages (MΦs) over 3 days in Roswell Park Memorial Institute (RPMI) medium containing 10% Fetal Bovine Serum (FBS) and 1% penicillin–streptomycin. On day 4, MΦs were pretreated for 18 h with dimethyl sulfoxide (DMSO) Phosphate-Buffered Saline (PBS) and IRAK4 inhibitor (IRAK4i) (1 µM, Sigma, SAINT LOUIS, MO #PZ0327; dose was based on our previous studies) (30, 32, 35) in serum-free RPMI. The IRAK4 inhibitor was prepared as a DMSO stock and diluted into culture media to a final DMSO concentration of 0.05%, which was used for both the inhibitor-treated and vehicle control groups. Thereafter, cells were stimulated with MSU (29) (100 µg/mL; Sigma #U2875) for 24 h and underwent enzyme-linked immunosorbent assay (ELISA) (Protein) and qRT-PCR (mRNA) analyses.

#### HepG2 cells

HepG2 was generously provided by the Khetani Lab, Department of Biomedical Engineering, University of Illinois. HepG2 cells (passage 9) were cultured in 10% Dulbecco's Modified Eagle's Medium (DMEM) and 1% penicillin–streptomycin. Confluent cells were seeded in 24-well plates and pretreated for 18 h with DMSO (PBS) and IRAK4i (1 µM, PF06650833, Sigma #PZ0327) in DMEM (without FBS). Thereafter, cells were stimulated with xanthine (2.5 mM; Sigma #X7375; dose was titrated as shown and previous studies) (36) for 24 h for mRNA analysis.

### Real-time RT-PCR

RNA was isolated using TRIzol and was reverse transcribed to cDNA using the RevertAid RT Reverse Transcription Kit (Thermo Scientific, Foster City, CA). SYBR green gene expression master mix (Bio-Rad, Waltham, MA) was used to perform qRT-PCR. Data were normalized with GAPDH and are presented as fold changes in RNA levels compared to control treatment, calculated following the 2^−ΔΔCt^ method.

### ELISA for cytokine analyses

Conditioned media from the macrophage, pretreated with IRAK4i (1 µM) overnight, followed by stimulation with MSU for 24 h, were collected, and cytokine levels of IL-1β, IL-6, IL-8, IL-10, TNF-α, IL-18, and TGF-β were measured using DuoSet ELISA kits (R&D Systems, Minneapolis, MN, USA).

### *In vitro* phagocytosis assay

The phagocytic activity of macrophages isolated from PBMCs was assessed using the Vybrant™ Phagocytosis Assay Kit (Thermo Fisher Scientific, Waltham, MA™). Briefly, macrophages (1 × 10^4^) were seeded in a 96-well flat-bottom plate, pretreated with IRAK4i overnight, and stimulated with MSU for 2 h. The culture medium was then replaced with 100 µL of the prepared fluorescent BioParticles suspension, followed by incubation at 37°C for 2 h. After incubation, the BioParticles suspension was removed, and the cells were washed twice with PBS. Subsequently, 100 µL of prepared Trypan Blue suspension was added and incubated for 1 min, and the fluorescence intensity was measured using a plate reader with ~480-nm excitation and ~520-nm emission, following the manufacturer’s instructions.

### Immunoblotting

Human macrophages were pretreated with IRAK4 inhibitor; the next day, cells were stimulated with MSU (100 µg/mL; Sigma #U2875) for 30 min. Cells were lysed in Radioimmunoprecipitation Assay buffer (RIPA) buffer, and protein was measured using a BCA method (Pierce™ BCA Protein Assay Kit, Thermo Fisher Scientific, Lenexa, KS, USA). Equal amounts of protein (25 μg) were loaded and separated by Sodium Dodecyl Sulfate (SDS)–polyacrylamide gel electrophoresis and transferred onto a nitrocellulose membrane (Bio-Rad, CA, USA). Blots were probed using rabbit polyclonal antibodies specific for p-p38 (4511S), p-JNK (9251S), p-ERK (4370S), and p-NFκb (3033S) (1:1,000, Cell Signaling, Danvers, MA) and GAPDH (2118S) for equal loading (1:3,000, Cell Signaling). Blots were then incubated with IRDye^®^ 680RD Goat anti-Rabbit IgG Secondary Antibody (926-68071) (1:10,000, LI-COR, Lincoln, NE) and IRDye^®^ 800CW Donkey anti-Mouse IgG Secondary Antibody (926-32212) (1:10,000, LI-COR) for 2 h, and protein bands were visualized using Odyssey CLx Imaging System (LI-COR).

### In-Cell Western

In-Cell Western (ICW) was performed to quantify the relative levels of phosphorylation of IRAK4 in PBMCs following treatment as mentioned earlier. Briefly, macrophages were seeded in 96-well plates at a density of 1 × 10^4^ cells per well. Cells were then pretreated with IRAK4 inhibitor overnight and stimulated with MSU for 30 min. Following treatment, cells were fixed with 4% paraformaldehyde and permeabilized using 0.1% Triton X-100. Cells were blocked for 2 h with Intercept^®^ (PBS) Blocking Buffer (927-70001, LI-COR) and incubated with primary antibodies against p-IRAK (411927S), IRAK4 (4363S), and tubulin (3873S) overnight at 4 °C. After being washed with Phosphate-Buffered Saline with Tween (PBTS), cells were incubated with IRDye-labeled secondary antibodies (LI-COR) for 1 h at room temperature in the dark. Fluorescent signal was detected using an Odyssey CLx Imaging System (LI-COR).

### Statistical analysis

For the experiments involving comparison of more than two groups, one-way ANOVA was performed with p < 0.05 as the confidence interval. Pairwise comparisons were performed using Šídák’s *ad hoc* test with a confidence interval of 95% (p < 0.05).

## Results

### TLRs and cytokines associated with gout flare and remission

TLRs contribute to the initiation and progression of inflammation in arthritic diseases by recognizing harmful stimuli and activating immune pathways that lead to the production of pro-inflammatory cytokines and mediators. To explore the role of TLRs and key cytokines in gout pathogenesis, we reanalyzed a publicly available dataset (34), focusing on the expression of TLRs, IL-1β, IL-18, TNF-α, IL-10, and TGF-β in patients experiencing gout flare versus remission.

**Figure 1:**
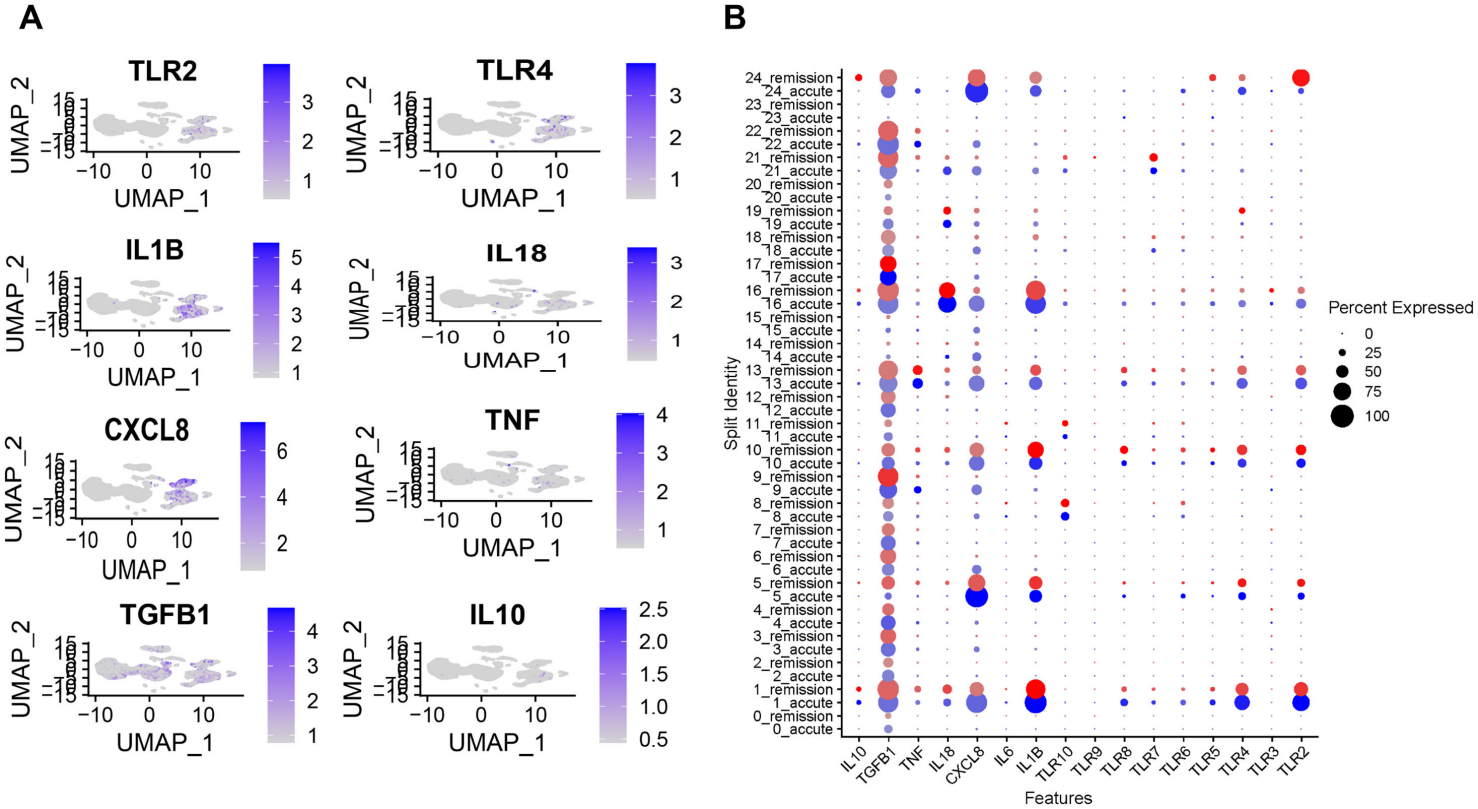
TLR2 and TLR4 are associated with the severity of gout. **(A)** Uniform Manifold Approximation and Projection (UMAP) plot showing distinct clustering of cell populations in patients with gout flare and remission phase. **(B)** Expression levels of TLRs and cytokines in gout flare and remission. Dot plot represented by color gradient, with gout flare depicted by blue and remission shown in red. TLR, Toll-like receptor.

Our analysis revealed that TLR2 and TLR4 expression was significantly reduced during remission compared to the flared state, whereas other TLRs exhibited similar trends without substantial changes (**Figures 1A, B**). Consistent with reduced TLR activation, the expression of IL-1β and IL-18, key inflammatory cytokines of the IL-1 family, also decreased during remission, while TGF-β, an anti-inflammatory cytokine, was upregulated. Together, these findings suggest that TLR2 and TLR4 are key drivers of IL-1 family cytokine signaling in gout and may play a central role in disease exacerbation.

### Inhibition of IRAK4 disrupts MSU-induced cytokine production

**Figure 2:**
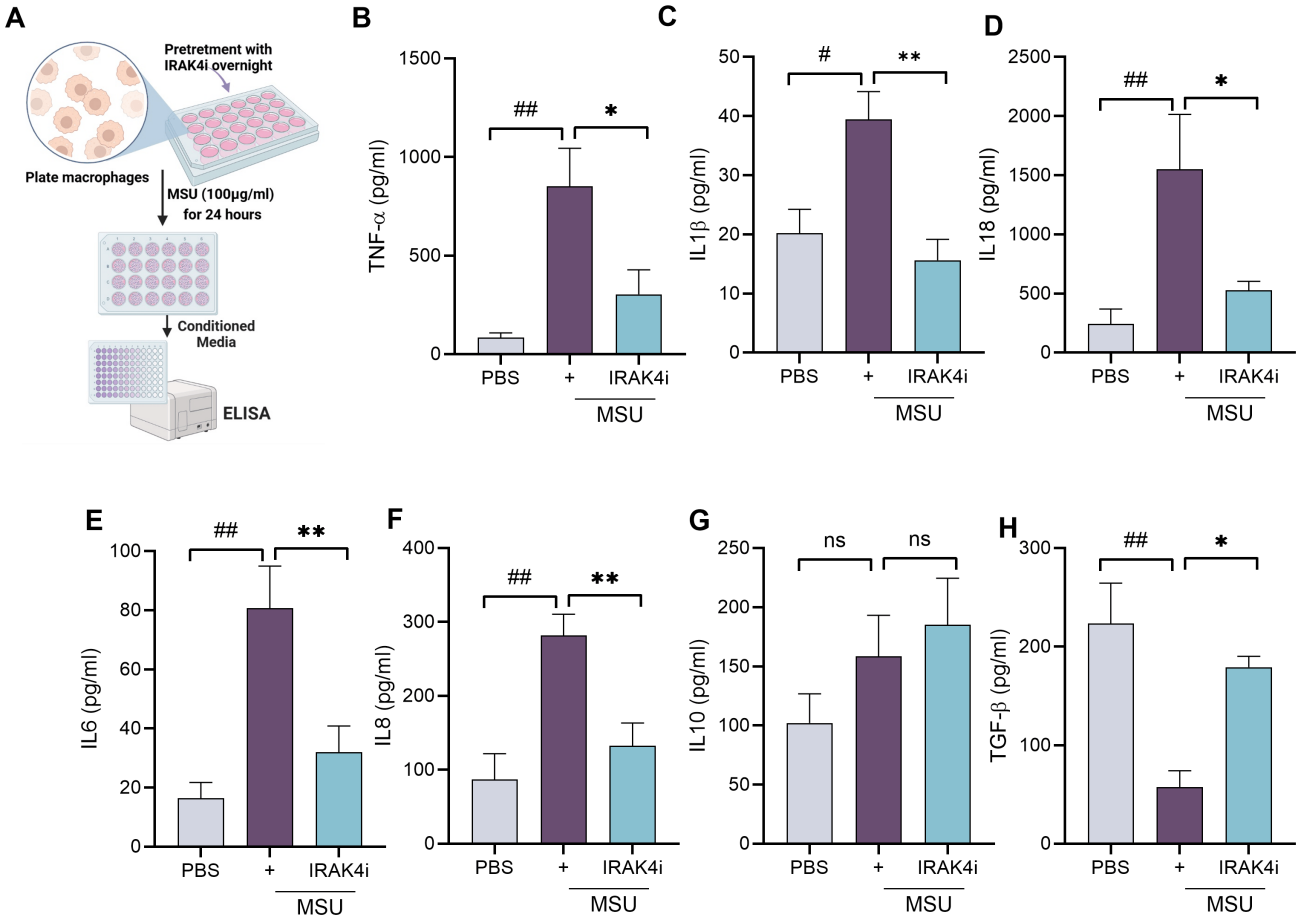
IRAK4 inhibition reduces MSU-induced pro-inflammatory cytokine production in human macrophages. **(A)** Schematic diagram showing experimental design from isolation of monocytes from PBMCs and their differentiation into macrophages, pretreatment with IRAK4i (1 µM) overnight, and stimulation with MSU (100 µg/mL) for 24 (h). **(B–H)** Conditioned media were utilized for quantifying cytokines such as IL-1β, TNF-α, IL-6, IL-8, IL-18, IL-10, and TGF-β secretion by ELISA. n = 5–6. The data are shown as mean ± SEM, ^#^ represents p < 0.05, ^##^ denotes p < 0.01 as compared to PBS, * represents p < 0.05, and ** denotes p < 0.01 as compared to MSU. Significant differences were determined by one-way ANOVA following Šídák’s multiple comparison test. IRAK4, interleukin-1 receptor-associated kinase 4; MSU, monosodium urate; PBMCs, peripheral blood mononuclear cells.

As TLRs play a role in gout, as shown in **Figure 1**, targeting downstream components of TLR signaling presents a promising therapeutic strategy. IRAK4, a key kinase in the TLR/IL-1 receptor pathway, mediates the activation of NF-κB and MAPKs, leading to the production of pro-inflammatory cytokines such as IL-1β and IL-18. We investigated the inflammatory response triggered by MSU crystals in human monocyte-derived macrophages and delineated the mechanism by which IRAK4 inhibition modulates this response. Notably, MSU exposure led to a robust increase in IL-18 secretion, followed by elevated levels of TNF-α, IL-6, IL-1β, and IL-8 (**Figures 2B–F**); a decrease in TGF-β; and no change in IL-10 (**Figures 2G, H**). Treatment with an IRAK4i significantly attenuated MSU-induced cytokine production by approximately 50%–70% and increased TGF-β by 70%.

**Figure 3:**
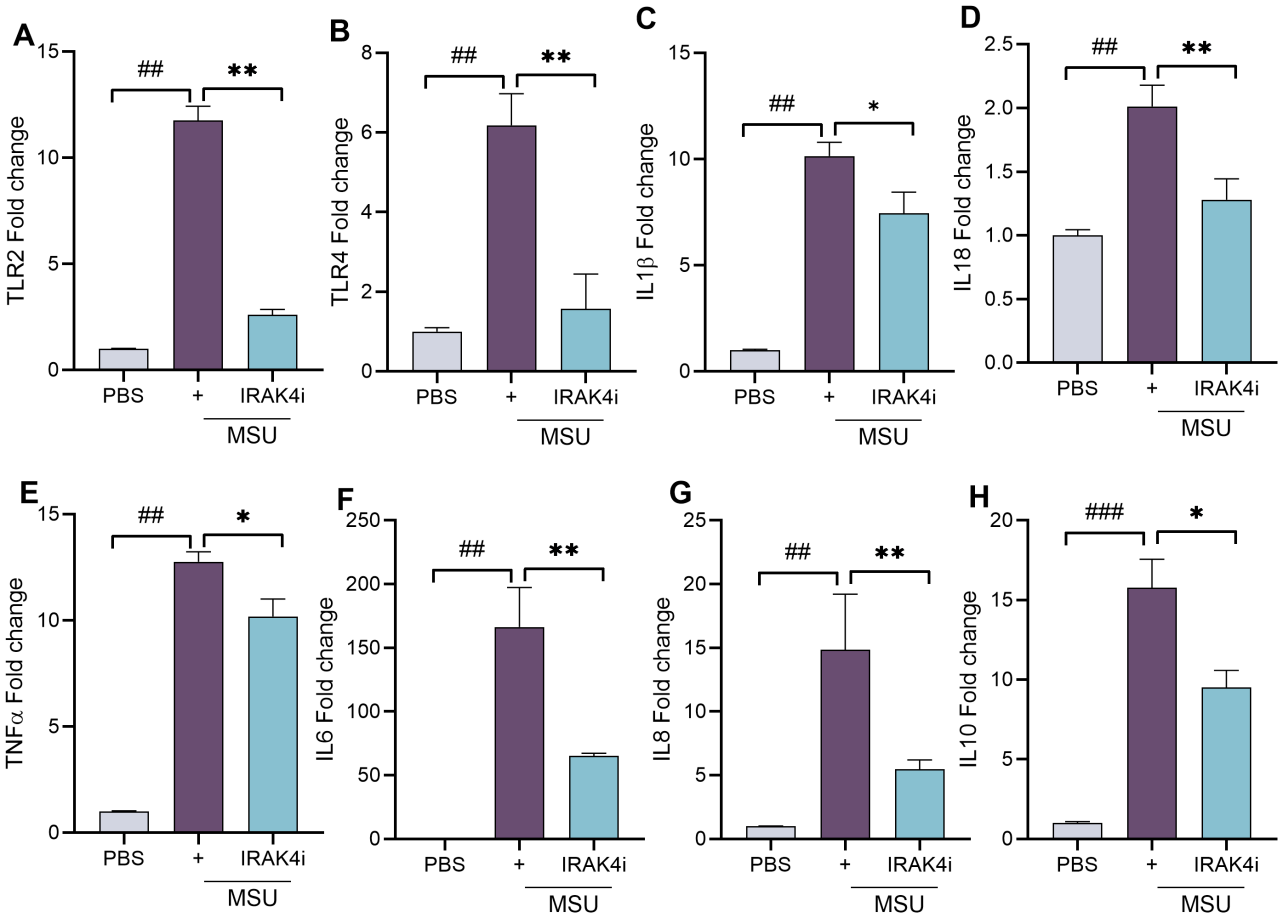
IRAK4 inhibition attenuates MSU-induced expression of inflammatory genes in human macrophages. **(A–H)** Monocytes were isolated from PBMCs and differentiated into macrophages with similar experimental conditions as stated in **Figure 3A**. Cells were harvested for RNA isolation and mRNA expression of TLRs (TLR2 and TLR4) and cytokines (IL-1β, TNF-α, IL-6, IL-8, IL-18, and IL-10) using real-time RT-PCR, n = 5–6. The data are shown as mean ± SEM, ^#^ represents p < 0.05, ^##^ denotes p < 0.01 as compared to PBS, * represents p < 0.05, and ** denotes p < 0.01 as compared to MSU. Significant differences were determined by one-way ANOVA following Šídák’s multiple comparison test. IRAK4, interleukin-1 receptor-associated kinase 4; MSU, monosodium urate; PBMCs, peripheral blood mononuclear cells; TLR, Toll-like receptor.

At the transcriptional level, IRAK4i markedly suppressed the expression of TLR2 and TLR4, reducing their induction by 70%–80% (**Figures 3A, B**). Furthermore, MSU stimulation strongly upregulated IL-1β, TNF-α, IL-8, IL-18 (~10–15 fold), and IL-6 (~150-fold), all of which were significantly suppressed (by 40%–60%) following IRAK4 inhibition (**Figures 3C–G**). We also observed an increase in IL-10 levels following MSU stimulation, which may represent a compensatory response by macrophages to counteract the inflammatory environment. Collectively, these findings demonstrate that IRAK4i effectively reverses the MSU-induced inflammatory phenotype, primarily by suppressing TLR signaling in human monocyte-derived macrophages.

### Inhibition of IRAK4 disrupts MSU-mediated phagocytic activity and suppresses NF-κB

**Figure 4:**
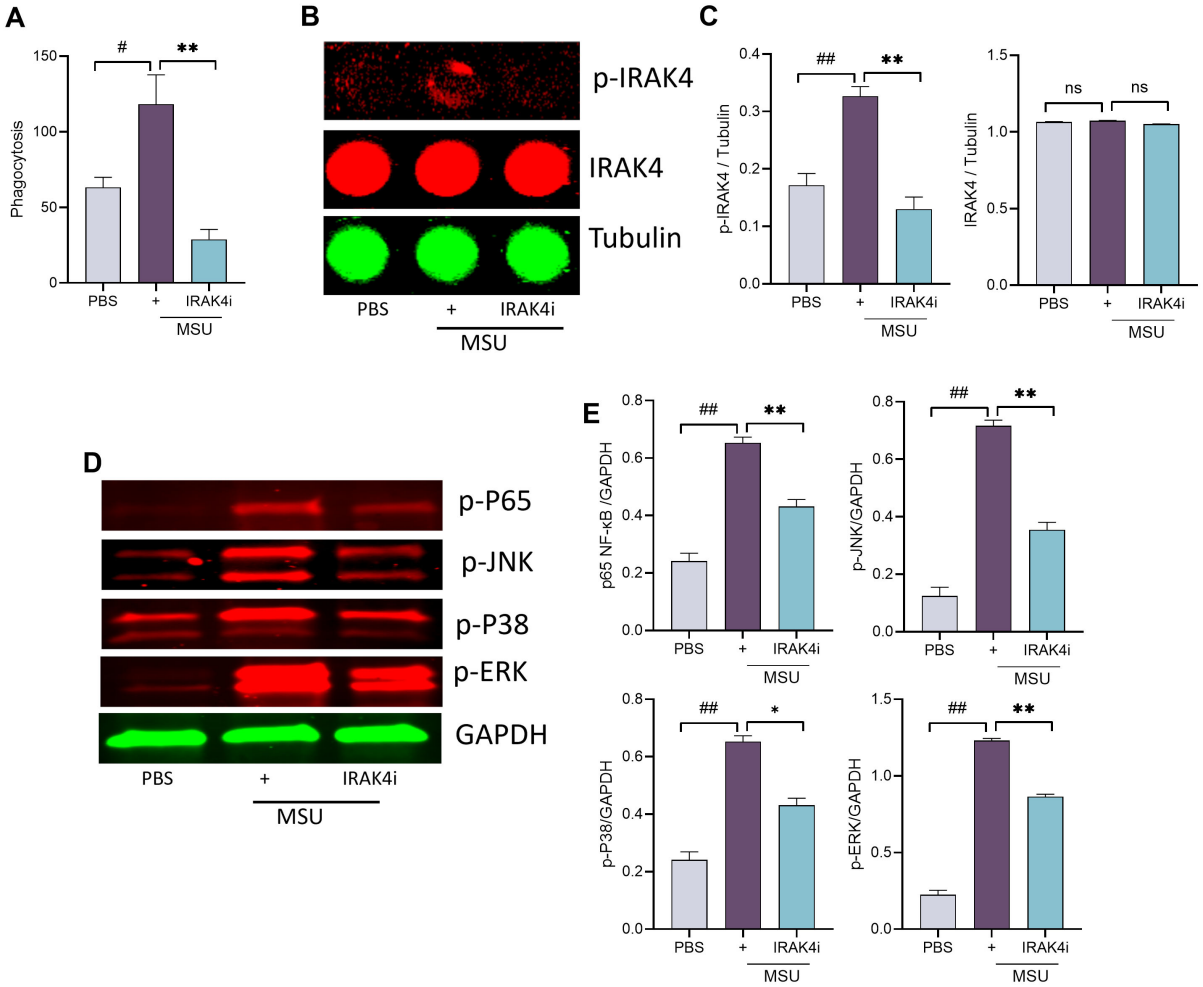
IRAK4 inhibition impairs MSU-induced phagocytosis and suppresses NF-κB signaling. **(A)** Macrophages were seeded into 96-well plates, incubated overnight with IRAK4i (1 µM), and stimulated with MSU (100 µg/mL) for 2 h; and absorbance was taken following manufacturer’s instructions. **(B, C)** For In-Cell Western, macrophages were seeded into 96-well plates (black), incubated overnight with IRAK4i (1 µM), and stimulated with MSU (100 µg/mL) for 30 min. Cells were fixed, blocked, and incubated with primary (p-IRAK4, IRAK4, and tubulin, 1:200) and secondary antibodies. Images were taken in LI-COR and analyzed. **(D, E)** Macrophages were seeded into 6-well plates, incubated with IRAK4i, and stimulated with MSU (100 µg/mL) for 30 min. Lysates were probed for p-ERK, p-p38, p-JNK, and p-NF-kB(p65) (1:1,000, Cell Signaling) and normalized to GAPDH (1:3,000, Cell Signaling), n = 3. Western blotting density was analyzed using ImageJ. The data are shown as mean ± SEM, ^#^ represents p < 0.05, ^##^ denotes p < 0.01 as compared to PBS, * represents p < 0.05, and ** denotes p < 0.01 as compared to MSU. Significant differences were determined by one-way ANOVA following Šídák’s multiple comparison test. IRAK4, interleukin-1 receptor-associated kinase 4; MSU, monosodium urate.

Phagocytosis is a key driver of inflammation in gout. Upon engulfing MSU crystals, macrophages activate TLR and downstream signaling pathways, leading to the release of pro-inflammatory cytokines such as IL-1β and IL-18 and amplifying inflammation via NF-κB activation. Our results demonstrated that IRAK4 inhibition effectively blocked MSU-induced phagocytosis in macrophages (**Figure 4A**).

To further investigate the mechanism, we examined whether IRAK4i affects IRAK4 phosphorylation and downstream NF-κB signaling. Exposure of human myeloid cells to MSU activated IRAK4 and led to phosphorylation of MAPKs (p38, ERK, and JNK), culminating in NF-κB pathway activation (**Figures 4B–E**, **Supplementary Figure S1**). Notably, IRAK4i suppressed MSU-induced activation of both IRAK4 and MAPK signaling in these cells. These findings suggest that targeting phagocytosis and its downstream effectors, particularly IRAK4, is an effective strategy to interrupt the inflammatory cascade triggered by MSU.

### IRAK4 inhibitor attenuates xanthine-stimulated cytokine response in HepG2 cells

In gout, dysregulation of the uric acid cycle can negatively impact the liver, as it is a major site of purine metabolism, where xanthine oxidoreductase (XDH) converts hypoxanthine and xanthine into uric acid. Moreover, elevated xanthine and uric acid levels can stimulate cytokine production in hepatocytes (e.g., HepG2 cells). Thus, in gout, the overactive uric acid cycle may burden the liver with both metabolic and inflammatory stress, exacerbating disease beyond the joints.

**Figure 5:**
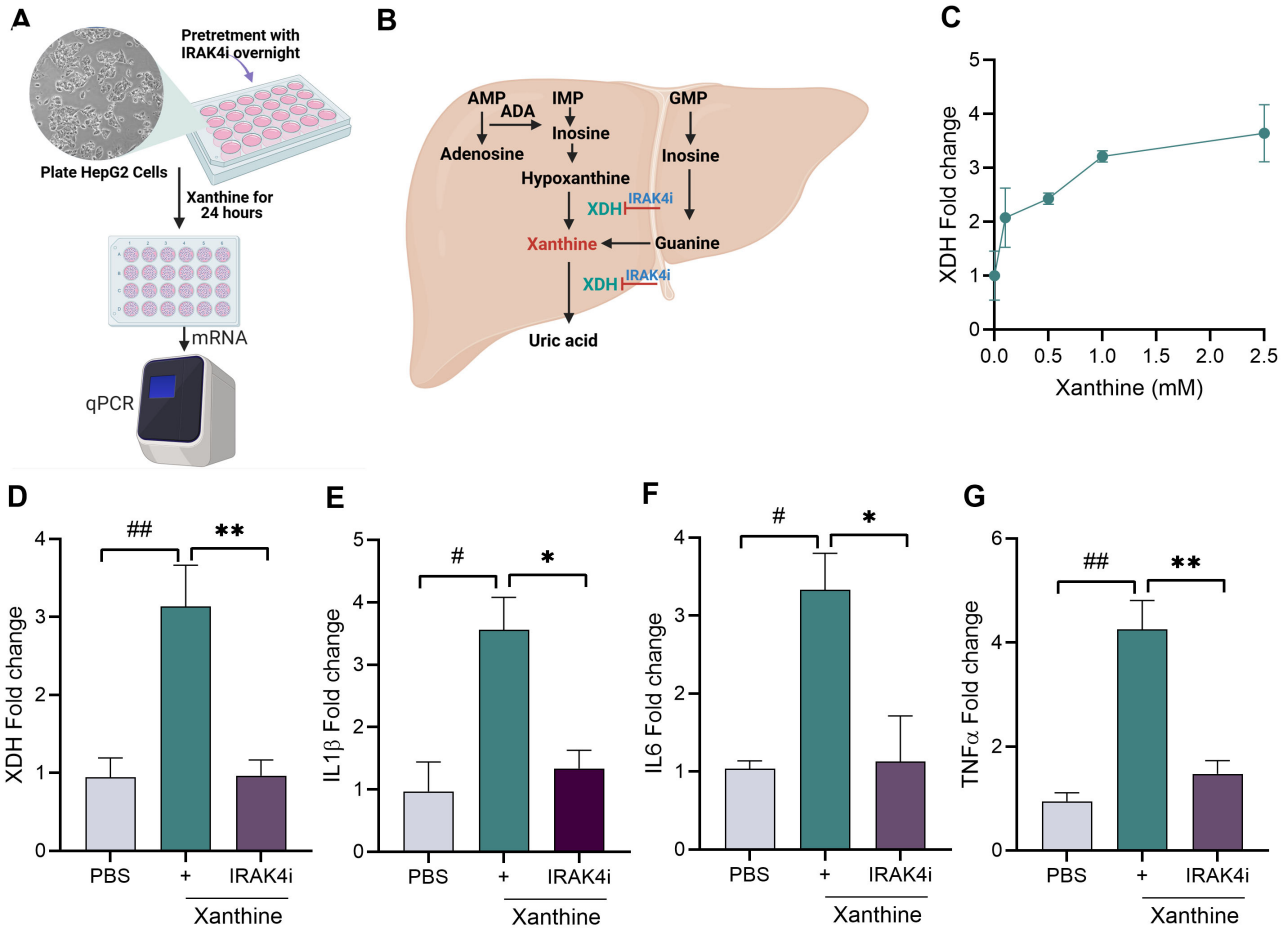
IRAK4 inhibition reduces xanthine-induced XDH and pro-inflammatory cytokine expression in HepG2 cells. **(A–C)** Schematic diagram showing experimental design; HepG2 cells were seeded in 24-well plates and stimulated with xanthine (0–2.5 mM), and expression of XDH was evaluated using real-time RT-PCR. **(D–G)** HepG2 cells were seeded in 24-well plates, pretreated with IRAK4i (1 µM) overnight, and stimulated with xanthine (2.5 mM based on dose-dependent study) for 24 h. Cells were harvested for RNA isolation and mRNA expression of XDH and cytokines (IL-1β, TNF-α, and IL-6) using real-time RT-PCR, n = 3. The data are shown as mean ± SEM, ^#^ represents p < 0.05, ^##^ denotes p < 0.01 as compared to PBS, * represents p < 0.05, and ** denotes p < 0.01 as compared to xanthine. Significant differences were determined by one-way ANOVA following Šídák’s multiple comparison test. IRAK4, interleukin-1 receptor-associated kinase 4; XDH, xanthine oxidoreductase.

We further tested whether IRAK4i has any benefits in suppressing the inflammation induced by xanthine in the liver. We took HepG2 cells, activated them with different doses of xanthine (0–2.5 mM), and observed the expression of XDH. Our results showed an increase in XDH expression dose dependently (**Figure 5C**). Furthermore, we treated HepG2 with IRAK4i (1 µM) overnight and stimulated with xanthine for 24 h. The expression of XDH and pro-inflammatory cytokines was increased, while IRAK4i inhibited their expression significantly, which showed the effect of IRAK4i not only on macrophages but also on HepG2 cells (**Figures 5 D–G**).

## Discussion

Over the past decade, multiple studies—including our own—have underscored the critical role of IRAK4 in regulating inflammation. While much of the research has focused on its involvement in conditions such as rheumatoid arthritis (30, 32, 37–39), psoriatic arthritis (31, 40), COVID-19 (35, 41–44), epilepsy (45), acute myeloid leukemia (46), and acute lung injury (47–49), its role in gout has remained largely unexplored. This study addresses that gap by investigating the effect of IRAK4 inhibition in an *in vitro* gout model. Our findings suggest that IRAK4 is a key mediator of inflammation triggered by MSU. The inhibition of IRAK4 significantly suppressed MSU-induced inflammatory signaling via the TLR–NF-κB pathway while also reducing xanthine-induced pro-inflammatory cytokine production and XDH expression in HepG2 cells. These results highlight IRAK4’s central role in gout-associated inflammation and its broader anti-inflammatory potential beyond immune cells.

Prior studies in knockout mouse models have demonstrated that TLR2 and TLR4 are key receptors recognizing MSU crystals (18, 20, 33, 50). Consistent with these findings, we observed that MSU stimulation increased TLR2 and TLR4 transcription in human PBMCs, while treatment with IRAK4i effectively suppressed their upregulation, suggesting that IRAK4 inhibition interferes with MSU-driven TLR signaling. IRAK4 inhibition significantly attenuated the MSU-induced inflammatory response, as evidenced by reduced expression of IL-1β, IL-18, IL-6, IL-8, and TNFα at both the transcriptional and protein levels.

Uric acid, a common cellular metabolite, in its crystalline form as MSU, is released from dying or damaged cells (51). The enhanced phagocytosis of MSU crystals is strongly linked to increased gout severity, as their uptake by neutrophils and macrophages triggers a robust inflammatory response (52–55). Notably, IRAK4 inhibition markedly suppressed MSU-induced phagocytosis in human macrophages, indicating its role in dampening early inflammatory triggers. To further elucidate the underlying mechanisms, we examined the phosphorylation status of key proteins in the NF-κB signaling pathway. Our results showed that IRAK4i significantly reduced the phosphorylation of these signaling molecules, thereby downregulating NF-κB pathway activity and limiting the downstream pro-inflammatory response in macrophages.

Given the liver’s central role in purine metabolism and uric acid production, and to evaluate the broader anti-inflammatory effects of IRAK4 inhibition beyond immune cells, we utilized HepG2 hepatocyte-like cells as an *in vitro* model. Earlier studies showed the activation of IRAKs in HepG2 treated with palmitic acid (56). HepG2 cells were stimulated with xanthine to mimic metabolic stress observed in gout (57, 58). Our findings showed that xanthine stimulation upregulated XDH and pro-inflammatory cytokines, whereas IRAK4 inhibitor treatment significantly suppressed both XDH expression and pro-inflammatory cytokine production, indicating that IRAK4 signaling may also contribute to liver inflammation (59, 60). These results suggest a potential role for IRAK4 inhibition in mitigating systemic and hepatic inflammatory responses linked to uric acid dysregulation.

**Figure 6:**
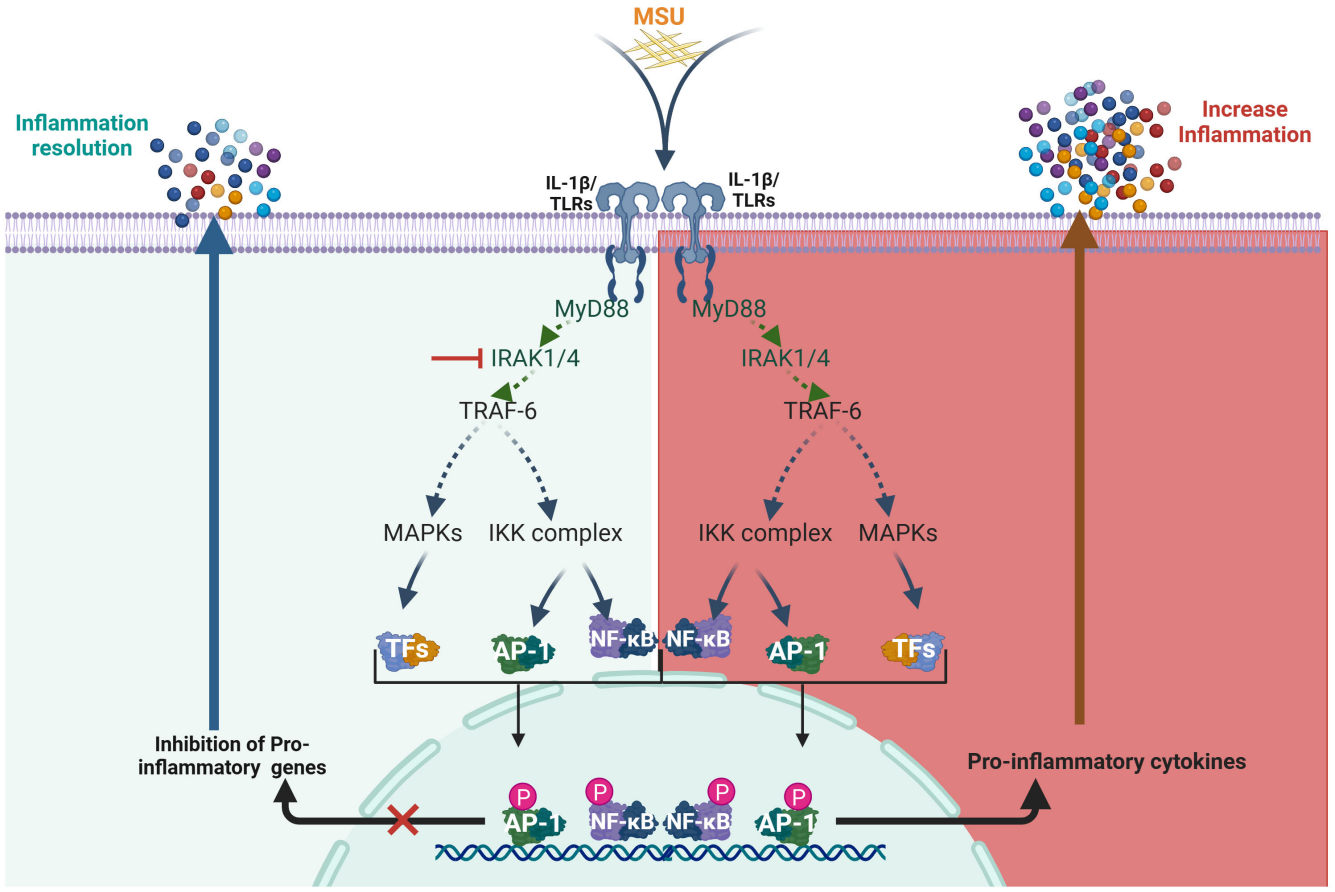
Schematic representation of the mechanism by which IRAK4 inhibition modulates MSU-induced inflammation. IRAK4, interleukin-1 receptor-associated kinase 4; MSU, monosodium urate.

Although clinical trials have demonstrated the therapeutic potential of IL-1 inhibitors like anakinra and, more recently, rilonacept, their limitations in effectively managing gout have also been recognized (61–64). These challenges highlight the need for novel, targeted therapies that not only address current shortcomings but also offer more practical delivery options. Our study demonstrates that the inhibition of IRAK4 signaling significantly reverses the pro-inflammatory profile induced by MSU crystals in human monocyte-derived macrophages. By disrupting key components of the TLR–NF-κB signaling axis (**Figure 6**), IRAK4 inhibition reduces the expression of critical cytokines such as IL-1β, IL-18, IL-6, IL-8, and TNFα. These findings not only reinforce the central role of IRAK4 in mediating gout-associated inflammation but also suggest that pharmacological targeting of IRAK4 may offer a promising therapeutic strategy to control excessive inflammation in patients with gout. One limitation of the present study is that we did not incorporate animal models of gout to validate our findings *in vivo*. We recognize the importance of such studies. Future experiments will focus on animal models of gout, where different delivery mechanisms at site-specific and systemic levels will be evaluated for dose response, efficacy, and toxicity.

## Data Availability

The original contributions presented in the study are included in the article/**Supplementary Material**. Further inquiries can be directed to the corresponding authors.

## Supplementary material

Supplementary Figure 1 Raw western blots of **Figure 4**.
