# Supplementary figures and images for: Modulation of IRAK4 as a therapeutic strategy against monosodium urate- and xanthine-induced inflammation in macrophages and HepG2 cells
Source: Front Immunol. 2026 Jan 8;16:1744393. doi: 10.3389/fimmu.2025.1744393 (PMC12823803; doi:10.3389/fimmu.2025.1744393)

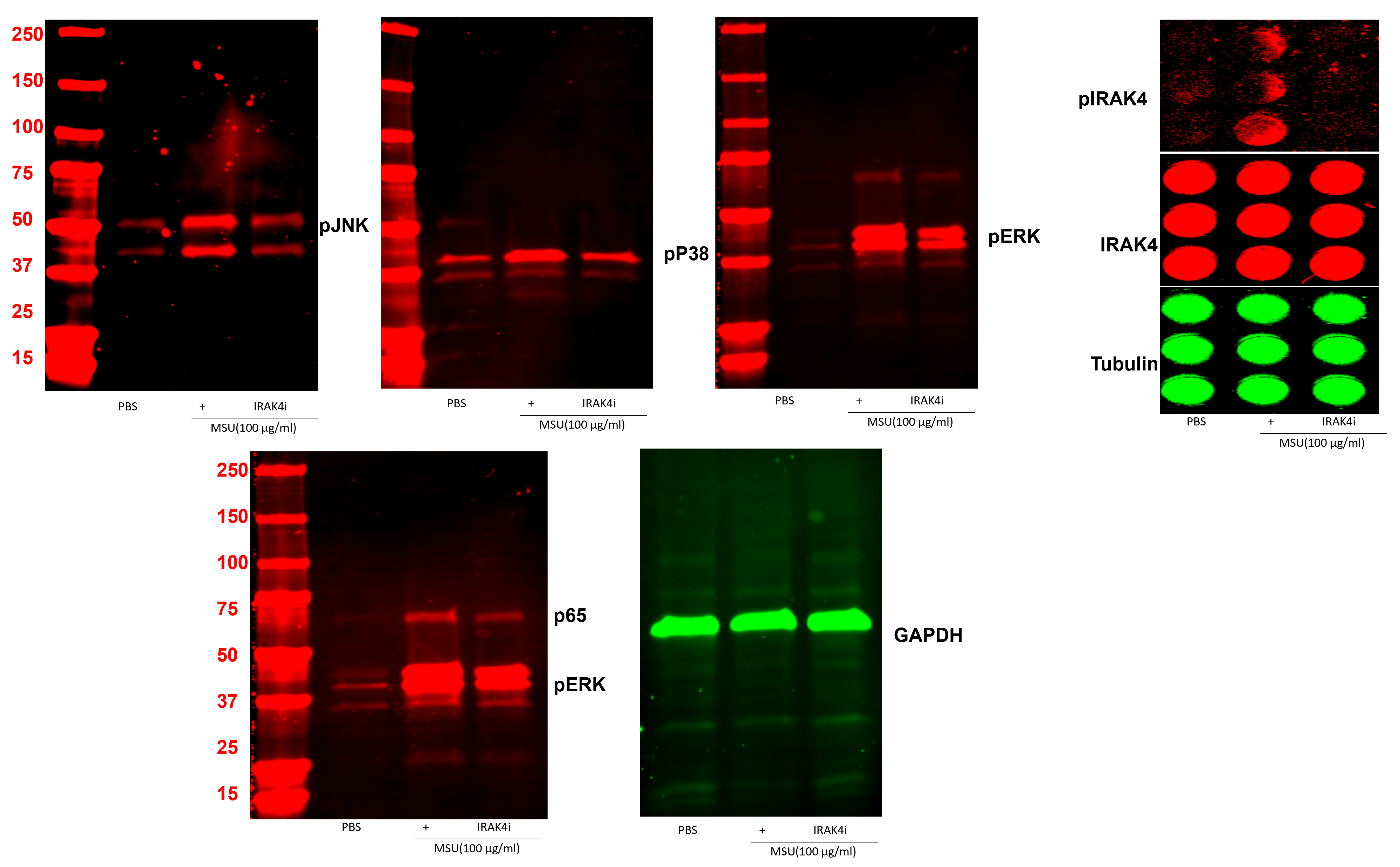

Supplement: Supplementary Figure 1 — Raw western blots of Figure 4. [file Image1.tif]
